# The Potential Role of Contraction-Induced Myokines in the Regulation of Metabolic Function for the Prevention and Treatment of Type 2 Diabetes

**DOI:** 10.3389/fendo.2017.00097

**Published:** 2017-05-02

**Authors:** Brian P. Carson

**Affiliations:** ^1^Health Research Institute, Physical Education and Sport Sciences, University of Limerick, Limerick, Ireland

**Keywords:** exercise, myokines, muscle, endocrine, diabetes

## Abstract

Skeletal muscle represents the largest organ in the body, comprises 36–42% of body weight, and has recently been recognized as having an endocrine function. Proteins expressed and released by muscle that have autocrine, paracrine, and endocrine bioactivities have been termed myokines. It is likely that muscle contraction represents the primary stimulus for the synthesis and secretion of myokines to enable communication with other organs such as the liver, adipose tissue, brain, and auto-regulation of muscle metabolism. To date, several hundred myokines in the muscle secretome have been identified, a sub-population of which are specifically induced by skeletal muscle contraction. However, the bioactivity of many of these myokines and the mechanism through which they act has either not yet been characterized or remains poorly understood. Physical activity and exercise are recognized as a central tenet in both the prevention and treatment of type 2 diabetes (T2D). Recent data suggest humoral factors such as muscle-derived secretory proteins may mediate the beneficial effects of exercise in the treatment of metabolic diseases. This mini-review aims to summarize our current knowledge on the role of contraction-induced myokines in mediating the beneficial effects of physical activity and exercise in the prevention and treatment of T2D, specifically glucose and lipid metabolism. Future directions as to how we can optimize contraction-induced myokine secretion to inform exercise protocols for the prevention and treatment of T2D will also be discussed.

## Introduction

Skeletal muscle has recently been identified as an endocrine organ that synthesizes and secretes proteins known as myokines (1). These myokines are involved in autocrine regulation of metabolism in the muscle itself and paracrine/endocrine regulation of other tissues and organs such as the liver, adipose, and brain.

As skeletal muscle represents the largest organ in the body, the influence of myokines on whole-body metabolism is potentially significant (2, 3). As skeletal muscle contraction is likely the primary stimulus for myokine synthesis and secretion, it is plausible that myokines mediate, in part at least, beneficial adaptations to tissues in response to exercise. Recent research has identified several hundred myokines, a large sub-population of which are specifically induced by contraction (4). However, the specific bioactivity of a vast number of myokines remains largely undescribed and poorly understood. Furthermore, little is known about the role of type, intensity, or frequency of contraction in regulating myokine production and release.

Exercise has long been established as a central tenet to both the prevention and treatment of type 2 diabetes (T2D) (5). Though a number of mechanisms through which exercise confers these metabolic benefits have been well characterized (5), the pluripotency of exercise is not yet fully understood. One such mechanism is *via* cross-talk between tissues stimulated by contraction and release of myokines regulating tissue function. This creates a clear link between exercise and the regulation of whole-body metabolism. There have been several examples of this in recent research, most notably, the role of the contraction-induced myokine IL-6 in mediating skeletal muscle glucose uptake (6–8). These findings generated excitement as to the potential roles of contraction-induced myokines in the prevention of insulin resistance and metabolic diseases such as obesity and T2D. To date, a number of contraction-induced myokines have been identified which play a role in regulating glucose uptake, insulin sensitivity, and fat metabolism, leading factors in the development of T2D (9).

The purpose of this mini-review is to discuss known metabolic roles for contraction-induced myokines that aid in the prevention/treatment of T2D. Future directions in optimizing exercise protocols to maximize the potential of contraction-induced myokines by the type and intensity of exercise and how this informs exercise prescription will also be discussed.

## Myokines and Metabolism

Contraction-induced myokines have been shown to have autocrine, paracrine, and endocrine effects on numerous tissues. In this section, the evidence of contraction as a stimulus for myokine secretion, based on electrical pulse stimulation (EPS) models and/or an increase in circulating concentrations immediately post-exercise, and their effect on metabolic functions affecting the development of T2D in muscle, adipose, and liver will be discussed.

### Myokines Regulating Glucose Metabolism

#### IL-6

Evidence exists for a number of contraction-induced myokines with roles for glucose uptake and insulin sensitivity. IL-6 is most prominent in the literature and has been the focus since the early 2000s of those trying to identify the “exercise factor” through which skeletal muscles communicate to central and peripheral organs (10). IL-6 transcription in skeletal muscle and release to circulation in large volumes in response to contraction was first characterized in 2002 (11). Increased circulating concentrations of IL-6 are known to be affected by both the intensity and duration of contraction in humans (8, 12). Higher intensity and longer duration exercise result in increased circulating concentrations of IL-6 in humans (8, 12). IL-6 release in response to exercise is also dependent on the energy status of the cell, determined by pre-exercise glycogen content, whereby low glycogen content results in a greater release of IL-6 to the energy crisis in the muscle cell during contraction (6). *In vitro* studies demonstrate that IL-6 treatment increases glucose uptake through AMP-activated protein kinase [adenosine monophosphate kinase (AMPK)] and phosphatidylinosotol 3-kinase (PI3K) pathways (13). Carey et al. (7) reported increased insulin-dependent glucose uptake *in vivo* in response to IL-6 infusion. By contrast, Harder-Lauridsen et al. (14) found no increase in glucose uptake during euglycemic hyperinsulinemic clamp with IL-6 infusion in T2D individuals, though there was a reduction in the plasma insulin suggesting increased insulin sensitivity (14). Jiang et al. (15) found a differential effect of IL-6 treatment on primary myotubes from normal glucose tolerant and T2D, suggesting a blunted role of IL-6 on T2D muscle. IL-6 treatment upregulated both insulin-dependent and -independent glucose uptake and glycogen synthesis in healthy myotubes, but this effect was lost in T2D myotubes. This suggests that from a glucose control perspective, the contraction-induced myokine IL-6 is effective in the prevention of T2D but may be ineffective for glucose uptake in patients with existing T2D.

#### IL-13

IL-13 is released from human primary myotubes *in vitro* and has been demonstrated to have an “insulin-like” effect on glucose metabolism in human muscle by increasing glucose uptake, glycogen synthesis, and glucose oxidation in normal and T2D primary myotubes (15). This “insulin-like” effect is mediated through activation of Akt and PI3K pathways. IL-13 expression is increased in response to strength training in human skeletal muscle (16), but no evidence exists for an increase in plasma IL-13. This suggests the influence of IL-13 on glucose metabolism is localized to the muscle in an autocrine/paracrine manner.

#### Follistatin-Like-1 (FSTL-1)

Follistatin-like-1 is a secretory myokine of the follistatin family, known to be secreted *in vitro* by C2C12s (murine cell line) (17). Furthermore, Görgens et al. (17) demonstrated FSTL-1 expression and release from human primary myotubes. Interestingly, contraction of primary myotubes by EPS did not induce the secretion of FSTL-1; however, an increase in circulating plasma FSTL-1 in humans is observed following an acute bout of aerobic exercise. *In vitro* incubation of L6 myotubes (rat cell line) in FSTL-1 has been shown to increase glucose uptake in an AMPK- and calcium–calmodulin kinase-dependent manner (18), resulting in increased GLUT4 mRNA expression and translocation to the plasma membrane mediating enhanced glucose control.

#### Chitinase-3-Like-1 Protein (CHI3L1)

Electrical pulse stimulation of primary human skeletal muscle cells increases CHI3L1 expression and secretion (19). Acute aerobic and resistance exercise increase circulating CHI3L1; however, combined training had no effect, suggesting a transient exercise response. Evidence indicates that CHI3L1 regulates myoblast proliferation, suggesting a role in muscle growth thus affecting the size and volume of this organ as a “sink” for blood glucose (19). Furthermore, though CHI3L1 is induced by inflammation as well as contraction, it improves glucose uptake and insulin action under pro-inflammatory conditions in human primary skeletal muscle cells through activation of its receptor protease-activated receptor 2 (19). This suggests that CHI3L1 could regulate skeletal muscle glucose uptake under pro-inflammatory conditions observed in obesity and T2D.

#### IL-15

IL-15 is a known contraction-induced myokine secreted in humans post both aerobic and resistance exercise (20, 21) with similar responses between lean and obese participants (22). IL-15 has an effect on glucose uptake in C2C12 skeletal muscle cells (murine cell line) *via* activation of AMPK (23). Krolopp et al. (24) found a similar increase in glucose uptake with IL-15 treatment, mediated by an enhanced GLUT4 translocation to the plasma membrane. However, in contrast to the findings of Gray and colleagues, GLUT4 translocation was not initiated by activation of AMPK, but rather through the Janus kinase–signal transducer and activation of transcription protein 3 (STAT3) pathway. It is not entirely clear why there is no increase in phosphorylation of AMPK in this study, when using a higher dose of IL-15 (100 vs 1 ng/ml).

#### IL-8

IL-8 is secreted by primary human myotubes following EPS (25) and circulating IL-8 increases in response to endurance exercise in humans (26, 27). IL-8 is primarily associated with inflammation and angiogenesis; however, Gray and Kamolrat (23) demonstrated *in vitro* an increase in glucose uptake in C2C12s in response to treatment with IL-8 *via* phosphorylation of AMPK. A role for IL-8 in glucose uptake *in vivo* is less clear but may be mediated by increased vascularization, an effect which is lost in muscle from T2D (28).

#### Fibroblast Growth Factor-21 (FGF-21)

Fibroblast growth factor-21 treatment improves glucose tolerance and insulin sensitivity in the liver of obese Zucker rats (29). FGF-21 treatment has also been demonstrated to lower blood glucose and enhance insulin sensitivity in a diabetic mouse model (30). FGF-21, mediated by activation of Akt, improves glucose uptake in primary human adipocytes, which is enhanced when combined with insulin, reducing the required level of insulin to achieve the same glucose uptake (31). Muise et al. (32) confirmed reduced plasma glucose in WT, HFD, and diabetic mouse models treated with FGF-21 perfusion and identified upregulation of genes associated with several pathways such as glucose uptake, and insulin receptor signaling regulated by FGF-21 in brown and white adipose tissue (WAT) and adipocytes *in vitro*. FGF-21 also increased basal and insulin-stimulated glucose uptake in primary human myotubes by increasing GLUT1 mRNA and translocation to the plasma membrane (33). Circulating concentrations of FGF-21 are increased after an acute bout of endurance exercise in humans (34) and enhanced by higher intensity exercise (35). Short-term training also resulted in increased circulating FGF-21, which was associated with lower fasting glucose (36). Conversely, 3 weeks of sprint interval training results in reduced circulating FGF-21 (37). Similarly, 3 months of combined resistance and aerobic training resulted in a modest decrease in serum FGF-21 in obese women (38). This suggests that an acute bout of exercise leads to a transient increase in FGF-21 but the effect of chronic training is equivocal. Circulating FGF-21 is increased in T2Ds compared to normal glucose tolerant individuals and correlated with fasting insulin and BMI (33). Perhaps, the effect of chronic training is to decrease fasting insulin and adipose mass and thereby reduce circulating FGF-21. The acute increase in FGF-21 post-exercise is likely from muscle with the action of sensitizing muscle, adipose, and liver to insulin to facilitate glucose uptake.

#### Irisin

Irisin is a controversial candidate, primarily thought to be secreted not only by muscle but also in small amounts by adipose tissue. The main point of contention has been the detection of this myokine in its glycosylated and deglycosylated forms [for review, see Ref. (39)]. Future research should focus on detection by mass spectrometry as per (40); however, the *in vivo* data reported here use the best validated ELISA technique (39). Circulating irisin increases in response to high-intensity interval exercise, resistance exercise, and continuous moderate exercise in both healthy and metabolic syndrome patients (41). Some data suggest a greater increase following resistance compared with aerobic exercise (42). Serum irisin is regulated by exercise intensity, with greater increases following high-intensity exercise (43, 44). By contrast, other research reports an increase in the expression of FNDC5 in human skeletal muscle following 12 weeks of training but a paradoxical decrease in circulating irisin (45). Though synthesized in muscle, it is not clear if irisin is secreted from muscle directly either *in vitro* or *in vivo*. Incubation of L6 myotubes in irisin *in vitro* results in increased glucose uptake in a dose-dependent manner and is mediated by activation of AMPK and ACC (46). Irisin treatment also upregulates expression of PGC-1α4, a specific isoform associated with muscle hypertrophy, in primary myocytes (47). This was accompanied by increased IGF-1 and decreased myostatin expression, suggesting it a role in regulation of muscle growth, thus providing a larger muscle mass to act as a sink for blood glucose. Irisin perfusion in HFD mice resulted in decreased fasting blood glucose and improved glucose and insulin tolerance (48). Furthermore, FNDC5 overexpression in obese and HFD mice led to increased serum irisin resulting in decreased serum fasting glucose and improved glucose tolerance and insulin sensitivity in HFD mice (48).

#### Brain-Derived Neurotrophic Factor (BDNF)

The effect of resistance training on circulating BDNF remains equivocal. Several studies report no change in BDNF after either acute or chronic resistance training (49–53). By contrast, Yarrow et al. (54) and Coelho et al. (55) report increased plasma BDNF after acute and chronic resistance training. Circulating BDNF increases after both acute and chronic aerobic exercise in healthy participants [for review, see Ref. (56)]. Though a dose response is not apparent, there is evidence to support a greater increase in circulating BDNF following high-intensity exercise (57, 58), although whether muscle was the direct source of BDNF remains unclear. BDNF mRNA expression is increased by contraction of skeletal muscle cells; however, there is no evidence to show BDNF is secreted by muscle cells following contraction (59). BDNF treatment reduces blood glucose in a diabetic rodent model (60). Yamanaka et al. (61) also found that chronic BDNF infusion improved glucose uptake and metabolism in BAT and muscle of rodents.

### Myokines Regulating Fat Metabolism

#### IL-6

IL-6 infusion stimulates lipolysis and whole-body fatty acid (FA) oxidation in healthy males (62). Similarly, IL-6 treatment in humans results in elevated FA oxidation measured by palmitate oxidation and disappearance rates and a decreased respiratory quotient, peaking 60 min post-infusion (63). Increased whole-body lipolysis is mediated by STAT3 signaling to upregulate skeletal muscle but not adipose tissue lipolysis. Similarly, Petersen et al.(64) found that IL-6 infusion resulted in an increased rate of palmitate appearance and disappearance in human serum of both normal glucose tolerant and T2D patients. *In vitro* experiments confirmed increased lipolysis in adipocytes and FA oxidation in L6 myotubes (60). These data suggest IL-6 plays a beneficial role in fat metabolism through the upregulation of lipolysis in skeletal muscle and an increase in FA oxidation that is maintained in T2D.

#### IL-15

IL-15 administration to rodents resulted in a 35% decrease in WAT and a 20% decrease in circulating triglycerides, suggesting a role for IL-15 in lipid metabolism (65). Overexpression and oversecretion of IL-15 in a transgenic mouse model resulted in decreased total body and visceral fat (66). Treatment of adipocytes with IL-15 resulted in decreased deposition of lipids (67). Pierce et al. (68) perfused human subcutaneous adipose tissue with IL-15 *via* a microdialysis probe and observed an increase in adipose tissue lipolysis of lean participants. However, this effect was lost in obese participants, whereby, IL-15 perfusion suppressed lipolysis. Interestingly, muscle-derived IL-15, induced by exercise did not have an effect on adipose tissue lipolysis in either lean or obese (68). Therefore, the role of IL-15 in regulating lipolysis in humans remains equivocal and requires further investigation. Little information exists on a role for IL-15 in lipid oxidation; however, Almendro et al. (69) demonstrated an effect of chronic IL-15 administration to rodents on the fate of an exogenous lipid bolus. IL-15 reduced *de novo* lipogenesis in adipose tissue in response to an exogenous lipid load and favored oxidation in muscle and liver *via* the upregulation of FA transport genes. Further evidence for IL-15 and lipid oxidation in both healthy and T2Ds is required.

#### Brain-Derived Neurotrophic Factor

Brain-derived neurotrophic factor treatment of L6 myotubes and intact *ex vivo* muscle results in increased FA oxidation *via* activation of AMPK (59). Chronic BDNF treatment reduces circulating FAs, total cholesterol, and phospholipids in a diabetic rodent model (60). Chronic intracerebroventricular BDNF administration is also shown to decrease body weight, fat mass, adipocyte size, and serum triglycerides and promote lipolysis (70). Exercise induced increases in plasma BDNF are equivalent in obese and non-obese individuals but are not associated with increases in either whole-body glucose or FA oxidation (71). Further work is required to determine the effect of contraction-induced BDNF on fat metabolism in muscle, adipose, and liver.

#### Irisin

Irisin treatment of 3T3-L1 adipocytes *in vitro* induces increased gene expression of lipolysis-related genes including adipose triglyceride lipase, hormone-sensitive lipase (HSL), and protein expression of fatty acid-binding protein 4, suggesting irisin has potential to increase lipolysis (72). By contrast, Wang et al. (73) found no effect of irisin on HSL or ATGL protein expression or expression of lipolysis-related genes in 3T3L-1 adipocytes. Irisin perfusion in HFD mice resulted in decreased serum cholesterol, triglycerides, and free FAs (48). FNDC5 overexpression in obese and HFD mice led to increased serum irisin resulting in decreased serum triglycerides and free FAs in obese and HFD mice (48). Irisin treatment of adipocytes resulted in increased expression of UCP-1 and increased energy expenditure. Irisin also induced expression of metabolic genes (CPT-1, PPARα, HSL) and prevented lipid accumulation (74). Irisin treatment of myocytes also elevated FA oxidation suggesting a protective effect against progression of T2D (75).

#### Myonectin

Myonectin, a member of the C1q/TNF-related protein family, is expressed in skeletal muscle and released to the circulation in response to exercise in animal studies (76). *In vivo* myonectin administration reduced circulating levels of free FAs without altering adipose tissue lipolysis in mice. This reduction in circulating free FAs is purported to occur by an increase in FA uptake upregulated by increased expression of FA transport genes such as CD36, FATP1, Fabp1, and Fabp4 (76).

#### Fibroblast Growth Factor-21

Fibroblast growth factor-21 treatment of 3T3L-1 adipocytes attenuates lipolysis and expression of perilipin (77) and has also been shown to increase hepatic FA oxidation (78). Chronic FGF21 treatment reduces serum and hepatic triglyceride levels in diet-induced obese mice (79). These data suggest an influential role for FGF-21 in regulation of lipid metabolism.

## Optimizing the Myokine Response for the Prevention and Treatment of T2D

This review has outlined the role of myokines in regulating glucose and fat metabolism as potential mechanisms through which exercise can protect against the onset or progression of T2D. To harness the actions of contraction-induced myokines, we must establish the types, intensity, and volume of contraction required to maximize these regulators to inform future exercise protocols for the prevention and treatment of T2D. Table 1 summarizes what we currently know with respect to the contraction-induced myokines discussed, in terms of how they are modulated by exercise and their actions in metabolic regulation. The role for aerobic exercise is clear, with evidence for an increase in circulating concentrations post-exercise for all myokines discussed, except IL-13, which appears to be acting in an auto/paracrine manner in response to resistance training only. This aligns with current recommendations that aerobic exercise is the primary component of any regimen in the prevention/treatment of T2D (80). It is logical to expect a dose response to contraction; but so far, few studies have demonstrated an effect or a minimum duration of exercise (12, 27). Similarly, few studies have demonstrated an effect for intensity, with higher intensity exercise generally eliciting a greater increase in circulating myokines (8, 35, 41, 43, 44, 57, 58). Resistance exercise effectively enhances circulating concentrations of the majority of myokines discussed (Il-6, IL-15, BDNF, CHI3L1, irisin) confirming the rationale for inclusion in prevention/treatment protocols.

**Table 1:** **Summary of known myokine response to contraction and metabolic action**.

Myokine	Secreted from muscle	Electrical pulse stimulation	Increase in plasma/serum	Aerobic exercise	Resistance exercise	Exercise duration effect	Exercise intensity effect	Glucose uptake	Glucose oxidation	Lipolysis	Lipid oxidation	Pathway
IL-6	✓	✓	✓	✓	✓	✓	✓	↑	↑	↑	↑	AMPK, PI3K, STAT3
IL-8	✓	✓	✓	✓		✓		↑				AMPK
IL-13	✓			✓	✓			↑	↑			Akt, PI3K
IL-15	✓		✓	✓	✓			↑		↕	↑	AMPK, JAK–STAT3
BDNF	✓		✓	✓	✓		✓	↑		↑	↑	AMPK
CHI3L1	✓	✓	✓	✓	✓			↑				CHI3L1/PAR-2, p44/42, p38 MAPK, Akt
FGF-21	✓		✓	✓			✓	↑	↑	↕	↑	Akt, p44/42 MAPK
FSTL-1	✓	✓	✓	✓				↑				AMPK, CaMK
Irisin	✓		✓	✓	✓		✓	↑				AMPK
Myonectin	✓		✓	✓							↑	FA transport

*✓, positive evidence; ↑, evidence for an increase in metabolic action; ↕, evidence for both an increase and decrease of metabolic action; BDNF, brain-derived neurotrophic factor; CHI3L1, chitinase-3-like-1; FGF-21, fibroblast growth factor 21; FSTL-1, follistatin-like-1; AMPK, adenosine monophosphate kinase; PI3K, phosphatidylinositol 3-kinase; Akt, protein kinase B; JAK, Janus kinase; STAT3, signal transducer and activation of transcription protein 3; PAR-2, protease-activated receptor 2; MAPK, mitogen-activated protein kinase; CaMK, calcium–calmodulin kinase; FA, fatty acid*.

In order to optimize future exercise prescription and policy to maximize the response and effect of myokines on metabolism, it is clear from this mini-review that there is a need to definitively characterize the following in both healthy and T2D participants: (i) the myokine response to an acute bout of aerobic exercise of varying durations (as low as 10 min); (ii) the myokine response to aerobic exercise of varying intensities; and (iii) the myokine response to resistance exercise of varying volume and intensities. To date, much of the evidence describing the mechanism through which recently identified myokines modulate metabolic function have been characterized using *in vitro* cell models which do not necessarily translate to the *in vivo* human situation. Though this is a necessary preliminary approach, it is important to acknowledge this as a significant limitation when interpreting the findings of the current literature.

Finally, this review has focused predominantly on tissue crosstalk by myokines released to the circulation; however, it is likely that more myokines are secreted post-exercise exclusively to the interstitium where they are exerting a local effect. More work is required to identify the entire *in vivo* contraction-induced secretome by techniques such as interstitial microdialysis. Furthermore, there is a need to establish the bioactivity of contraction-induced myokines for both local and systemic tissues.

## Author Contributions

BC conceptualized the paper, reviewed the literature, and wrote the manuscript in its entirety.

## Conflict of Interest Statement

The author declares that the research was conducted in the absence of any commercial or financial relationships that could be construed as a potential conflict of interest.
